
JOURNAL INFORMATION
==============================
NLM Title Abbreviation: Insights Imaging
Journal ID: Insights Imaging
NLM Journal ID: 101532453

Insights into Imaging

EISSN: 1869-4101
Publisher: Springer

ARTICLE INFORMATION
==============================
PMCID: PMC10185715
PMID: 37188879
DOI: 10.1186/s13244-023-01419-4
Article ID: 1419
Article version: 1
Subjects: Meeting Abstracts

EuSoMII Annual Meeting 2022 ‘Your portal to AI’ Book of Abstracts

Electronic publication date: 2023 May 16
Publication date: 2023 May
Volume: 14
Issue: Suppl 1
Issue sponsor: Publication costs were funded by the European Society of Medical Imaging Informatics.
Electronic Location ID: 86
Copyright: © The Author(s) 2023
License: Open AccessThis article is licensed under a Creative Commons Attribution 4.0 International License, which permits use, sharing, adaptation, distribution and reproduction in any medium or format, as long as you give appropriate credit to the original author(s) and the source, provide a link to the Creative Commons licence, and indicate if changes were made. The images or other third party material in this article are included in the article's Creative Commons licence, unless indicated otherwise in a credit line to the material. If material is not included in the article's Creative Commons licence and your intended use is not permitted by statutory regulation or exceeds the permitted use, you will need to obtain permission directly from the copyright holder. To view a copy of this licence, visit http://creativecommons.org/licenses/by/4.0/.
License URL: https://creativecommons.org/licenses/by/4.0/

Conference name: EuSoMII Annual Meeting 2022 'Your portal to AI'
Conference location: Valencia, Spain
Conference date: 14-15 October 2022
issue-copyright-statement: © European Society of Radiology (ESR) 2023

==============================

### Insights into Imaging

Education and strategies in European radiology

EuSoMII Annual Meeting 2022 'Your portal to AI' Book of abstracts / Volume 14 / Supplement 1 / May 2023

Insights Imaging (2023) 14 (Suppl 1): 86
https://doi.org/10.1186/s13244-023-01419-4
Published: 16 May 2023

EuSoMII Annual Meeting 2022

Your portal to AI

Valencia, 14-15 October 2022

### SS 1 - BEST ORAL TALK

### Lifelong nnUNet: A framework for standardized medical continual learning

*Camila González, Amin Ranem, Daniel Pinto dos Santos, Ahmed Othman, Anirban Mukhopadhyay*

Short Summary: We present *Lifelong nnU-Net*, an open-source framework that places state-of-the-art continual segmentation at the hands of researchers and clinicians. Built on top of the nnU-Net and equipped with necessary modules for training and testing continual models, we ensure broad applicability.

Purpose/Objectives: Most research and product development in medical AI focuses on designing **static systems for a dynamic clinical world**. This disparity often leads to abrupt drops in performance post-deployment. Over time, as acquisition standards and patient populations change, models trained with an outdated data distribution become **unreliable**. *Continual learning* systems are raising the interest of medical image researchers and regulatory bodies alike. Despite recent advances, research on continual medical segmentation is still in its infancy. We introduce a standardized framework that allows for easy benchmarking and a fair comparison between methods.

Methods and materials: Five popular continual learning methods are implemented in *Lifelong nnU-Net*: Rehearsal, Elastic Weight Consolidation (EWC), Learning without Forgetting (LwF), Riemannian Walk (RW) and Modelling the Background (MiB). We evaluate them on prostate and hippocampus segmentation in MRIs over four and three open-source datasets (*tasks*), respectively, which vary in terms of subject population and acquisition conditions. For each anatomy, we train a patch-based 3D nnU-Net for 250 epochs per task. The nnU-Net automatically configures the architecture from the first task. For the continual methods, we select hyperparameters used in previous work or which showed reasonable loss trajectories.

Results: An effective approach should prevent *forgetting*, i.e. performance deterioration for early tasks, while maintaining sufficient *plasticity* to learn new knowledge. Rehearsal, which re-uses samples from previous tasks in later training stages, consistently performs best (maintaining a Dice of at least 85.94±0.76% for prostate and 88.17±3.63% for hippocampus across all tasks). However, this strategy cannot always be used due to privacy considerations. EWC and RW are good alternatives, reducing the amount of forgetting, though this often comes at the cost of plasticity loss. For instance, while the sequential model has a Dice of 91.91±0.38% on the last prostate task, the Dice decreases to 87.79±0.83% for EWC. For hippocampus segmentation this behaviour is more pronounced, with the Dice of the last task falling from 90.92±1.08% to 31.93±6.09% for EWC and 20.75±6.99% for MiB.

Conclusion: Our results shine light on the recent progress and open challenges of training segmentation models continuously. We hope that *Lifelong nnU-Net* accelerates R&D in this nascent yet impactful field.

Disclosure: No conflicts of interest to disclose.

Keywords: continual learning, lifelong learning, image segmentation

### SS 2 - BEST POSTER

### Automatic evaluation of printing accuracy and presence of defects in geometrically simple 3D printed objects based on micro-CT imaging

*Adrián Belarra, Irene Hernández-Girón, Margarita Chevalier*

Short Summary: Anthropomorphic phantoms attempt to mimic the size, shape, composition and radiation behaviour of the human body for quality control (QC) and assessment (QA) of medical imaging equipment as surrogates to some extent of patients. The development of 3D printing technology is a low-cost alternative that enables a fast and customised manufacture of such objects. QC of the printing processes requires a non-destructive analysis of the inner structure of samples that is possible using micro-CT.

Purpose/Objectives: To propose an automated micro-CT based framework for quality assessment of 3D printing process. The print accuracy and defect localisation in simple geometric objects to predict printing limitations of more complex objects and to compare performance of 3D-printers.

Methods and materials: A 10mm radius hollow sphere with 2mm wall thickness (S), a 20mm side hollow cube (C) with 2mm wall thickness and a 20mm high concentric-cylindrical-shells (CCS) object (4 shells of 1mm thickness shells and 2mm radius inner cylinder), were 3D-printed using PLA (BCN3D Sigma-R19; 0.3mm nozzle diameter; 0.1mm layer height). The objects were micro-CT imaged (Hamamatsu X-ray tube with 20μm focus size and 50μm pizel size flat-panel). A centring procedure based on opposing 2D-projections was created to avoid geometric misalignment artefact. An automated sample self-alignment method was developed and segmentation techniques were applied to the sample reconstructed volumes for structural characterization and measurement of air volume trapped between layers.

Results: A 14.3μm voxel size was achieved, obtaining the following results (average±standard deviation) expressed in milimiters. S: outer radius (10.22±0.02), inner radius (8.00±0.06). C: the outer/inner dimension were (20.00±0.05/16.00±0.03) along the deposition direction and (20.19±0.03/15.86±0.03), along directions on the printing bed surface. CCS: average thickness of shells were (1.06±0.12), cylinder radius (2.16±0.04). The air volume values were 39mm$^3$ for S, 13mm$^3$ for C and 58mm$^3$ for CCS. The number of layers and their average thickness were 194 layers of 0.11mm thickness for S, 195 layers of 0.10mm thickness for C and 190 layers of 0.11mm thickness layers for CCS.

Conclusion: An automated micro-CT based framework for non-destructive analysis of simple 3D printed objects was developed. Design-printed differences were compatible with nozzle diameter and layer height. This work is a first step based on simple objects, to standardize nondestructive quantitative QA methods of 3D printed anthropomorphic phantoms used in medical physics.

Disclosure: Irene Hernández-Girón has a personal grant from NOW (Veni-Talent Programme). Pr. Nr. 17378: through the eyes of AI: safe and optimal integration of Artificial Intelligence in the Radiology workflow.

Keywords: 3D printing; antropomorphic phantoms; quality assessment; micro-CT; self-alignment.

### SS 3 - RUNNER UP ORAL TALK

### The Ambivalence Paradox: How does attitudinal ambivalence affect radiologists' reliance on artificial intelligence (AI) suggestions

*Ferdinand Mol, Mohammad H. Rezazade Mehrizi*

Short Summary: Radiologists collaborating with AI-powered decision aids can fall for cognitive biases caused by inappropriate reliance on the AI. Current research suggests attitudinal ambivalence as a mitigator of inappropriate reliance, though its concomitant cognitive consumption can instead cause exacerbation of inappropriate reliance. To investigate this paradoxical effect, we conducted an online experiment where radiologists carried out mammography examinations together with input from AI, after having been primed on a univalent (positive or negative), ambivalent, or neutral attitude towards AI. The ambivalently primed participants were found to demonstrate more inappropriate reliance and less appropriate reliance than the neutral control group, highlighting the "double edged" nature of attitudinal ambivalence and suggesting a need for further research on it as an intervention.

Purpose/Objectives: To investigate the effect of attitudinal ambivalence on the cognitive biases that threaten the successful socio-technical implementation of AI decision aids.

Methods and materials: We conducted a 2*2 factorial experiment to examine how radiologists conduct mammography examinations depending on being primed to 1) be critical to AI's suggestions or not, and 2) to trust AI or not (where the ambivalent group was simultaneously primed to be both). All participants examined the same sequence of 15 mammographies, via an online interface where they could interact with the mammogram, check a heatmap, and ask for AI suggestions. We collected all click-based data, as well as the final decisions of the radiologists on the presented cases. Using a combination of descriptive analytics and variance analysis, occurrences of inappropriate (under- or over-reliance) and appropriate reliance were investigated.

Results: Participants primed to trust AI demonstrated more over-reliance and less appropriate reliance than the control group. Additionally, ambivalently primed participants demonstrated more inappropriate reliance and less appropriate reliance than the neutral control group. Those primed to be critical towards AI also demonstrated more under-reliance and less appropriate reliance. Those who were primed univalently spent more time on performing the examinations, and on their own analysis of the image before checking out the heatmap and AI suggestion, than those who were primed to become ambivalent and the control groups.

Conclusion: The findings highlight the necessity to be careful about generalizing the efficacy of attitudinal ambivalence for mitigating inappropriate reliance. Instead, they suggest the need for a deeper understanding of the impacts of attitudinal priming as an intervention.

Disclosure: None

Keywords: Artificial Intelligence, Automation Bias, Algorithmic Aversion, Reliance, Attitudinal Ambivalence, Cognitive Dissonance, Online Experiment, Mammography.

### SS 4

### Patient Satisfaction with a System for Lay-Language Annotation of Radiology Reports

*C.E. Kahn*

Short Summary: Patients using an EHR portal found it helpful to read radiology reports annotated with illustrations and lay-language definitions of medical terminology.

Purpose/Objectives: Radiology reports are intended to communicate information to healthcare professionals; the reports are typically difficult for patients to understand, especially for those with limited reading skills. Our institution has sought to improve patients' ability to understand their radiology reports, and has developed and integrated a system called PORTER (Patient-Oriented Radiology Reporter) into our health system's online patient portal. We sought to measure patients' satisfaction with the system.

Methods and materials: Of the 4651 concepts with lay-language definitions available the beginning of the current analysis, 52% of definitions were readable at the 4th-grade (age 10) level and 73% were readable at the 6th-grade (age 12) level. The glossary incorporated a total of 14 410 terms including lexical variants; for example, the concept "medial meniscus" included plural forms ("medial menisci") and adjectival forms ("medial meniscal"). We incorporated a single survey question into the system's display which asked if the pop-up definitions and images made it easier to read the report. Responses were provided on a Likert scale from 1 ("not at all") to 5 ("a lot"). Patients were surveyed from September 2021 through May 2022. This HIPAA-compliant study was IRB approved; informed consent was waived.

Results: The survey received 10 859 responses, of which 1604 (14.8%) were positive and 5343 (49.2%) were strongly positive.

Conclusion: Efforts to improve patients' ability to read their radiology reports can be received positively by patients: in summary, 64% of patients agreed or strongly agreed that the PORTER glossary improved their ability to read and understand their report. Health literacy is a social determinant of health (SDOH), and efforts that provide tools to address limited health literacy can support the goal of achieving greater health equity across the populations we serve.

Disclosure: No competing interest.

Keywords: Reporting, patient- and family-oriented care, EHR, patient portal, usability.

### SS 5

### Tracer: A multi-blockchain traceability system with applications in healthcare

Andy S Alic, J. Damian Segrelles Quilis, Pau Lozano, Sergio López-Huguet, Ignacio Blanquer

Short Summary: We propose a novel tracing system, *Tracer*, powered by blockchain technology. It allows secure storage of user actions (called traces) executed on a multi-service/multi-application platform for the creation of datasets from medical data and their usage in training AI models.

Purpose/Objectives: One of the most recent and impactful developments in trustworthy distributed systems in the IT field is the blockchain technology. It has applications in different domains, such as in healthcare where, due to the increase of digitalisation/tele-medicine/patient data sharing, the blockchain's inherent characteristics (e.g. security, traceability, and distributed nature of the underlying network) could make it a fundamental building block on ensuring the integrity of the data and the non-repudiation of the operations.

Methods and materials: *Tracer* is a web service that allows authenticated users (basic authentication and OpenID Connect using Keycloak) to add/read traces to/from one or more active blockchains. It supports multiple private/public blockchains (BigchainDB and Hyperledger Besu), each one with its own configuration, and one or more active at any moment. Our choice of the storage platform was driven by the excellent match between the technology's characteristics and the requirements of the project: tamper-proof, create-read only system that can be distributed to multiple partners (to verify each others' transactions easily). To mitigate the slower response inherent to the blockchain tech and to be sure that no trace is lost if the transaction containing it gets rejected, we implemented a caching feature to hold traces temporarily, until they are successfully added to the blockchain. *Tracer* does not store any personal or private user/patient information (in or with the traces), just anonymised (study identifiers) or hashed data (e.g. SHA256 of medical images).

Results: We already deployed *Tracer* on the CHAIMELEON platform to record the following user actions: dataset creation / metadata update / use on the platform, AI model creation / usage / publication on the platform's marketplace.

Conclusion: The blockchain technology has the potential to improve security, increase traceability, and efficiently distribute both the workload and the underlying stored data, as demonstrated by its use with *Tracer* on the CHAIMELEON platform.

Disclosure: No conflict of interest. *Tracer* developed as part of the CHAIMELEON EU funded project (DOI 10.3030/952172)

Keywords: medical imaging, traceability, blockchain, repository

### SS 6

### Deep learning-based tumour segmentation: from single-view 2D probability map prediction to multi-view averaged based ensembling

Alessia De Biase, Nanna M. Sijtsema, Johannes A. Langendijk, Peter M.A. van Ooijen

Short Summary: Medical image segmentation is one of the most studied fields of research in deep learning (DL). Medical images are endowed with valuable volumetric information and high dimensionality. However, 3D networks are not always the best fit because of high computational costs, inference time, and risk of overfitting due to the limited amount of annotated data. We designed a novel 2.5D deep learning (DL) segmentation model which generates slice-by-slice oropharyngeal tumour segmentation predictions on registered PET-CT images.

Purpose/Objectives: To assess the effect of multi-view averaging based ensembling fusing results from the three different orthogonal cross-sections and comparing it with each single-view performance.

Methods and materials: 138 oropharyngeal cancer patients (OPC) treated with (chemo)radiation in our institute were included. PET and CT images and primary tumour contours (GTVp) manually delineated by radiation oncologists were collected, which were used as ground truth.

The segmentation network was trained on 2D data made of three consecutive slices (one sequence) extracted from the axial (M-a), coronal (M-c) and sagittal (M-s) plane of concatenated PET-CT images. A 3-fold cross validation (113 patients) was performed on each cross-sectional view resulting in a total of 9 models trained for 150 epochs. During testing, predictions were obtained for 25 patients as probability maps. Since every slice is a part of three consecutive sequences, three probability maps were predicted per slice, which were averaged. In addition, averaging based ensembling was performed by averaging single-view and multi-view predictions.

No probability threshold was applied to the pixel values of the outputs. 2D mean dice score coefficient (DSC) was used to assess the model performance for different probability thresholds. Wilcoxon matched-pairs signed rank test was used to test the impact of multi-view averaging based ensembling.

Results: A significant difference was found between mean DSC results calculated on predictions obtained from single-view and the multi-view ensembling results (M-a, M-c: $p<0.001$, M-s: $p=0.003$). The multi-view ensembled predictions showed a reduced number of false positives derived from high predicted probabilities from M-s, while increased low predicted tumour probabilities matching the GTVp from M-a.

Conclusion: The model performance was influenced by high intensity regions on PET, especially when training on sequences extracted from the axial view. Multi-view averaging based ensembling mitigates this effect compared to single-view probability map predictions, showing to be beneficial for the final segmentation performance of the proposed DL framework in both cases where the tumour is highly visible on PET and not.

Disclosure: There is no conflict of interest to declare.

Keywords: deep learning; multi-view ensembling; probability map; tumor segmentation

### SS 7

### An open code solution for designing lung vessel phantoms: one step towards standardization of 3d printed anthropomorphic phantoms

*Irene Hernandez-Giron[1*], Sara de Scals Carmona[2], Julia Garayoa[3], Margarita Chevalier del Río[2]*
*Affiliations: [1]Division of image processing (LKEB), Radiology Department, Leiden University Medical Center (LUMC), Leiden, The Netherlands*
*[2]Departamento de Radiología y Medicina Física. Universidad Complutense de Madrid (UCM), Madrid, Spain*
*[3]Servicio de Protección Radiológica, Fundación Jiménez Díaz, Madrid, Spain*
**Contact info: irene.debroglie@gmail.com*

Short Summary: 3D printing is becoming a popular manufacturing technique for customized test objects in medical imaging, in particular for anthropomorphic phantoms. An open-code was developed for learning purposes to help medical physicists and other professionals to become familiar with mathematical anthropomorphic models of small vessel trees to be combined with 3d printing techniques.

Purpose/Objectives: To present a simple open code to generate customized models of small vessel tress that can represent to some extent lung or liver vessels, to spread the use of anthropomorphic modelling and 3D printing among medical physicists and other professionals.

Methods and materials: An in-house code for automatic generation of small vessel trees based on Matlab was developed. An interface allows the user to select the number of vessel generations, angles, radius and lengths, within certain constrains and also introduce such values as input in a table individually per vessel to create more realistic structures and design variability. A quick view option allows to visualize the vessels scaffolding and modify it on the fly. The final output of the software is an STL file, (input for a 3D printer of choice), and the table of input values for the vessel model. In this way each model has a paired "identity card" as design ground truth. The gold standard provided by the mathematical model can be compared to the printed objects or medical images of them to evaluate the printing process. Physical measurements (vernier caliper) can be performed on the small models to compare different printers, methods and materials.

Results: Small vessel trees were generated adapted to the user specifications, together with their design geometric characteristics. Phantoms were 3d printed (smallest vessels 1 mm diameter), selecting an HP multijet fusion 4200 printer and PA11 and TPU as materials. The phantoms were scanned in an Canon Aquilion Genesis system with a thorax protocol, showing attenuations in the range of lung vessels without contrast (-10 HU, 60HU range, respectively).

Conclusion: A simple and open code for modelling anatomical structures (vessels) to be combined with 3D printing techniques is presented for learning purposes and is accesible contacting the first author. The models generated in this code are not meant to be used for quality control of imaging systems or to represent accurately the human anatomy without further checking and development.

Disclosure: I.Hernandez-Giron has a personal grant from NWO Talent programma-Veni (Pr.17378: "Through the eyes of AI: safe and optimal integration of artificial intelligence in the Radiology workflow")

Keywords: 3d printing, anthropomorphic phantom, open code, computed tomography, image quality

### SS 8

### Classification for Radiogenomics to Predict the MGMT promoter methylation status in mpMRI scans

*Mostafa Karami, Mirjam Schöneck, Liliana Lourenco Caldeira*

Short Summary & Purpose/Objectives: Glioblastomas are the most aggressive forms of solid brain tumors of the central nervous system. The overall survival period is reported to be less than 15 months after diagnosis. MGMT gene silencing is a potentially proper predictive element in determining the mortality rate for glioblastoma patients undergoing chemotherapy. Analyzing the correlation between different image characteristics and MGMT promoter methylation status through ML tools could play an essential role in the automatic aided diagnosis approach. By doing data preprocessing, this study aims to extract features from public data provided by the RSNA and investigate this relationship with various classification methods.

Methods and materials: Using Pyradiomics 1153 features have been extracted from the original images, their segmented forms, Laplacian of Gaussian, and Wavelet filters to get features with more details. XGBoost classifier is used since it is desirable to reduce the number of features and identify the optimal number for the most relevant results. The model used nested cross-validation to obtain better outcomes by finding the best set of hyperparameters. This approach is also exclusively applied to different MRI sequence types to point out the importance of each for final user support.

Results: The final test results give the best F1-score for SVM (0.60) with 10 features. LR and MLP approaches chose 12 optimal features with lower F1-score (0.57 and 0.59). On average, T1 sequence type gives higher F1-scores compared to other sequence types. The optimal number of features increases in T1 for each method, and the standard deviation for SVM is the lowest (0.070). The optimal number of features is decreased significantly in T1Gd and T2 types.

Conclusion & Disclosure: This study demonstrated acceptable performance by the proposed feature extraction, feature selection methods, and ML classification algorithms. Although the deep learning approach would result in better performance metrics, considering insight into radiomics features and performing classification lead to valuable results for the final user. Undoubtedly, better results can be obtained by accessing more extensive data, which points out the importance of data quantity. Analyzing different optimal features also would be a good starting point for future research to focus on the most critical aspect of brain tumor MRI images.

Keywords: Feature extraction, MGMT Promoter, Classification, mpMRI, Segmentation, Radiomics

### SS 9

### External validation of an AI tool for multiple organ segmentation on CT images: performance vs training set size

*Anna Macula, Silvia Gonella, Sonia Colombo Serra, Marco Alì, Sergio Papa and Giovanni Valbusa*

Short Summary: a convolutional neural network was trained and optimized on CT-ORG public dataset for multi-organ segmentation. The performances were evaluated for different sizes of the training set on both internal and external validation datasets, collected from public and proprietary databases. The quality of the segmentation improves in precision and robustness with the training set's size, with acceptable results already including at least 50 annotated cases.

Purpose/Objectives: to evaluate the performance of a deep learning (DL) method for multi-organ segmentation trained on the CT-ORG database varying training set's size. External validation was carried out using a public database and data from our hospital (Centro Diagnostico Italiano (CDI).

Methods and materials: a 2D UNET architecture was trained on CT images from CT-ORG database (110 patients, 5 labels: liver, bladder, lung, kidneys and bones, resolution 1.44x1.44x1.44 $mm^3$) to perform multiple organ segmentation. Loss functions optimization (intersection over union (IOU), Dice Similarity Coefficient (DSC), categorical cross-entropy) and hyperparameters tuning were carried out using a validation set derived from the same database (internal validation). External validation was then performed on a sample set from Amos2022 challenge database and from a clinical study performed at CDI (10 patients each). DSC, IOU, precision, recall and specificity were applied as evaluation metrics.

Results: The reached DSC on the internal validation were 0.906, 0.841, 0.959, 0.867 and 0.869 for liver, bladder, lung, kidneys and bones, respectively. The performance gets worse up to 20% with the decrease of the training set from 100% to 10% (mean value lowered up to 20%, standard deviation increased up to three-fold). The same trend was confirmed by the external validation: DSC on CDI data were 0.934, 0.849, 0.959, 0.624 and 0.834 for liver, bladder, lung, kidneys and bones, respectively; DSC on Amos2022 challenge database were 0.800, 0.708, 0.912, 0.667 and 0.728 for liver, bladder, lung, kidneys and bones, respectively. These lower values may be justified by the different population in the test and training dataset (Chinese vs Caucasian), or by the great diversity of the data that characterizes Amos2022 database.

Conclusion: These results show that public databases are sufficient to train a multi-organ segmentation model to a level of accuracy which could be clinically useful also on locally acquired data. The increment of the size of the training set leads to significant improvement in precision and robustness, reaching satisfying results already when only 50 cases are included.

Disclosure:Sonia Colombo Serra and Giovanni Valbusa are employees of Bracco Imaging SpA, Marco Alì is a consultant of Bracco Imaging SpA. Research activities were sponsored by Bracco Imaging SpA.

Keywords: multi-organ, segmentation, deep learning, CT

### SS 10

### Compensating the impact of small patches on 3D U-Nets for precise segmentation of coronary vessels

*Jaroslaw Goslinski, Marcin Kostur, Filip Malawski, Mikolaj Stryja, Jakub Nalepa*

Short Summary: We tackle the problem of automated segmentation of coronary vessels in the coronary computed tomography angiography (CTTA) scans using U-Nets, and investigate the impact of the patch size on their performance. The spatial context captured within larger 3D patches is often critical for precise delineation of the regions of interest, hence increasing the patch should improve the CTTA segmentation quality. For coronary vessels, however, such large spatial neighborhood may not be useful (or may even be "harmful") to delineate their tiny endings. We showed that the U-Nets operating over $48^3$ patches and followed by our post processing routine offer more precise segmentation of the vessels' endings and can be more conveniently deployed in clinical hardware settings due to small memory requirements.

Purpose/Objectives: To investigate the impact of the patch size on the performance of U-Nets for segmentating coronary vessels in CTTA, and to enhance the capabilities of the U-Nets operating on significantly smaller patches through introducing effective post processing.

Methods and materials: We investigate different 3D patch sizes ($48^3$ and $160^3$, with the latter patch capturing 37× more voxels), and show how can we enhance the segmentation quality of the U-Nets trained over the $48^3$ patches through compensating the lack of larger context in post processing. Here, we filter out all connected components which are positioned farther than 150 voxels (radial distance) from the center of the heart, and have a volume smaller than 500 voxels. We exploit a dataset of 193 clinically-acquired CTTA scans (112 used for training, whereas the remaining 81 for testing).

Results: Utilizing the $160^3$ patches led to indeed better-performing models as quantified by mean Dice, precision and recall (0.78, 0.69, 0.90, respectively), when compared to the U-Nets trained over $48^3$ patches: 0.47, 0.32, 0.93. Our post processing substantially enhances the resulting segmentation maps for the $48^3$ models (0.74, 0.63, 0.92), and make them more competitive with those trained with the $160^3$ patches (0.80, 0.74, 0.89 after post processing).

Conclusion: U-Nets operating over $48^3$ patches followed by the proposed post processing offer more precise segmentation of the vessels' endings and can be more conveniently deployed in clinical hardware settings due to small memory requirements.

Disclosure: The authors declare that there is no conflict of interest. This work was supported by the National Centre for Research and Development (POIR.01.01.01-00-0664/16).

Keywords: Coronary vessels, segmentation, CCTA, U-Net, patch size
